# Package of interventions for harm reduction among women who inject drugs in Nigeria

**DOI:** 10.1186/s13690-025-01753-7

**Published:** 2025-11-13

**Authors:** Aisha Nantim Dadi, Olujide Olusesan Arije, Kene Eruchalu, Jennifer Anyanti, Omokhudu Idogho, Yamen Okonkwo, Peace Ikani

**Affiliations:** 1 Society for Family Health, Abuja, Nigeria; 2 Institute of Public Health, College of Health Sciences, Obafemi Awolowo University, Ile-Ife, Nigeria

## Abstract

**Introduction:**

Women who inject drugs (WID) in Nigeria face significant barriers to harm reduction services, including punitive policies, stigma, and gender-based violence, which increase their risk of HIV and hepatitis. This study presents proposed intervention models aimed at enhancing harm reduction services for WID in Nigeria.

**Methodology:**

This study employed a human-centered design approach to develop interventions for WID in Nigeria. The resulting intervention models were informed by qualitative assessments and stakeholder engagement, and were aligned with national health policies.

**Result:**

The key interventions developed include strategic advocacy to engage stakeholders in fostering supportive harm reduction policies, human rights campaigns to address gender-based violence and educate WID on their rights, and training outreach workers in wound management and naloxone administration. Additional measures include the Help Card initiative for reporting GBV and accessing support, the Speak Out intervention, and tailored harm reduction education on safe practices and available resources. A multi-pronged empowerment approach aims to enhance agency, while subsidized healthcare services reduce financial barriers to HBV vaccination and cervical cancer screening. Social protection initiatives integrate WID into safety nets, ensuring access to health insurance, child advocacy, and nutritional support.

**Conclusion:**

The package of interventions offers a promising, multi-dimensional approach to harm reduction for WID in Nigeria. Through human-centered design and stakeholder engagement, the interventions address both structural and individual barriers, creating scalable and sustainable strategies adaptable to regional contexts.

**Table:** 

Text box 1. Contributions to literature
• There is limited documentation of structured harm reduction interventions specifically designed for women who inject drugs (WID) in Nigeria.
• Existing harm reduction programs rarely address gender-specific needs, social protection, or structural barriers faced by WID..
• This manuscript presents a defined package of interventions co-developed with stakeholders to guide program design and policy.
• The intervention models offer a replicable framework for integrating legal, health, and empowerment services for WID in similar settings.

## Introduction

Women who inject drugs (WID) in Nigeria encounter numerous risks, including unsafe injecting practices such as syringe sharing and assisted injecting [1, 2], exposure to sexual and physical violence [3, 4], and limited access to female-responsive harm reduction services such as needle–syringe programmes (NSP) and opioid agonist therapy (OAT) [5, 6]. Socio-cultural barriers further compound these risks and include stigma in health facilities [2, 7], childcare and caregiving burdens [8], economic deprivation [9], housing instability [2], police harassment, and legal risks related to criminalization [9]. Recent research has demonstrated that gender-specific barriers significantly impede WID’s access to essential harm-reduction services [10, 11]. These barriers encompass pervasive social stigma, gender-based violence (GBV), and socio-cultural discrimination. Furthermore, research suggests that innovative strategies are crucial for mitigating health risks and improving outcomes among WID, given the complex interplay of structural and sociocultural challenges they face [12, 13].

In Nigeria, punitive drug policies, entrenched social norms, and criminalization of drug possession and use further marginalises WID and restricts uptake of services such as as needle and syringe programmes (NSPs) and opioid agonist treatment (OAT), contributing to increased rates of blood-borne infections [14]. In addition to legal and social barriers, there is a significant gap in the availability of gender-sensitive services that are responsive to the needs of WID. Many existing harm reduction programmes are designed with a one-size-fits-all approach and do not account for the specific experiences and responsibilities of women, such as childcare and domestic roles [15]. Peer-led interventions and community-based support have been proven to create safe spaces where WID feel respected and understood, thus improving service uptake [16–19]. These approaches can help bridge the gap between the healthcare system and women who might otherwise be reluctant to seek help.

Furthermore, there is an urgent need for the integration of harm reduction services into existing healthcare frameworks, which is essential for improving outcomes among WID. Comprehensive interventions that combine primary healthcare, harm reduction, and psychosocial support have proven effective in reducing the transmission of blood-borne infections and enhancing overall well-being [20]. Multi-component strategies have been shown to be more successful in addressing the complex needs of marginalised populations compared to isolated interventions [21]. Hence, by integrating harm reduction services with other health and social services, intervention packages can offer a more holistic approach that meets the diverse needs of WID in Nigeria.

This report presents a set of context-specific interventions for harm reduction among Nigerian WID, developed through a co-creation process using human-centered design (HCD) principles. Our goal is to describe a comprehensive intervention package aimed at reducing HIV and hepatitis infections among WID while ensuring efficient implementation. The report also addresses strategies for promoting the rights and social inclusion of WID through structured, pragmatic approaches.

### Methodology

We used a multiphase HCD approach to assess the needs of WID in Nigeria and develop targeted interventions to meet these needs. Human-centered design (HCD) is an iterative, participatory approach that engages end users and stakeholders to co-define problems and co-create, test, and refine solutions [22]. Our approach consisted of a baseline qualitative assessment, co-creation workshop, and national and state-level stakeholder engagements. The qualitative assessment aimed to identify the specific needs of WID in Nigeria and develop recommendations for addressing them. The assessment adopted a phenomenological approach and was conducted in Abia, Gombe, and Oyo States. Six focus group discussion (FGD) comprising a total of 60 WID (10 per FGD). Twenty-four in-depth interviews (IDI) were conducted with stakeholders including program implementers, community-based organization (CBO) leads, state HIV focal persons, law enforcement representatives, human rights officials, and clinicians. Topics explored in the FGDs included access to harm reduction services, preferred service locations, pregnancy-related services, service quality, and barriers to utilisation. The IDIs, on the other hand, focused on available interventions, service delivery mechanisms, referral systems, and challenges faced by service providers. The full findings of the qualitative research are published separately.

The findings from the qualitative research were used as conversation starter for a three-day co-creation workshop in which held in Federal Capital Territory, Abuja, Nigeria in January 2024. The workshop involved 24 participants: five women who inject drugs; three representatives of community-based organizations; six from the National Agency for the Control of AIDS (NACA), State Agencies for the Control of AIDS (SACA), the National AIDS and Sexually Transmitted Infections Control Programme (NASCP), and State AIDS and Sexually Transmitted Infections Control Programme (SASCP); one from the National Human Rights Commission; nine program implementers from Society for Family Health and partner organizations; and one co-creation design expert.. The workshop opened with a presentation of the qualitative research findings, which formed the foundation for subsequent design activities. The design activities featured structured group activities and plenary discussions. The techniques used are presented in Table 1 below in the sequence of use.

**Table 1:** Co-creation workshop activities and descriptions

Activity	Description
1. Affinity mapping and synthesis	Participants clustered key insights from research findings using sticky notes and flip charts to identify common themes
2. Developing “How might we” (HMW) questions	Brainstorming sessions generated problem-framing questions to guide innovative solutions
3. Rough solution brainstorming	Groups explored potential interventions through open-ended discussions by proposing solutions to the HMW questions
4. Deep dive into rough solutions	Participants analysed feasibility, implementation challenges, and potential impact, focusing on solutions to their HMW questions
5. Prioritisation of ideas	A prioritisation matrix was used to evaluate ideas based on feasibility, impact, alignment with project goals, ease of implementation, and expected time frames
6. Development of detailed programme descriptions	Selected ideas were refined into structured intervention programmes, outlining objectives, implementation steps, monitoring strategies, and sustainability plans
7. Ranking of interventions	Participants scored proposed interventions, ranking them based on their likelihood of success

The process led to the development of a package of nine WID-focused interventions. The full methodological design of the co-creation process is reported separately.

A national-level validation workshop was conducted in Abuja following the three-day co-creation workshop. This one-day session engaged national stakeholders to review preliminary findings and outputs, and to provide input for refinement of proposed interventions. Subsequently, separate state-level validation meetings were held in each of the three participating states. Feedback from these meetings was used to further revise the intervention components. In August 2024, a national dissemination meeting was convened with representatives from the study states, law enforcement agencies, implementing partners, NACA, UNODC, and other stakeholders. The research findings were presented and validated, providing final input into the co-creation process outputs. The final output of these processes was a set of nine interventions organized under three thematic categories as follows:

1. *Strategic Communication Interventions* – These interventions focus on influencing knowledge, attitudes, and behaviors through targeted messaging, advocacy, and community engagement. Their aim is to raise awareness, reduce stigma, promote rights-based practices, and mobilize support for policy and programmatic change.
2. *Empowerment and Harm Reduction Interventions* – These interventions are designed to enhance the agency of women who inject drugs (WID) by equipping them with knowledge, tools, and support systems to protect their health, assert their rights, and mitigate drug-related harms.
3. *Integrated Support and Health Interventions* – These interventions combine social welfare, economic empowerment, and health service access into a comprehensive support framework for WID. They aim to reduce structural barriers, improve health outcomes, and enhance the socio-economic stability of beneficiaries.

## Profile of interventions

This section outlines the proposed interventions, detailing all critical components necessary for their understanding, operationalisation, and implementation. The objective here is to present fully developed, implementation-ready intervention models.

### Strategic communication interventions

#### Strategic advocacy activity

##### Background

The Strategic Advocacy Activity addresses the limited institutional and policy support for harm reduction programs, which restricts their effectiveness and coverage. This lack of support undermines the provision of essential services to WID and compromises both their health outcomes and rights. The intervention is designed to secure the active engagement of key stakeholders to improve the implementation and long-term viability of harm reduction initiatives.

##### Program justification

Stakeholder engagement is necessary to improve access to harm reduction services for WID. Active support from relevant actors will enhance program implementation and secure the operational and institutional conditions required for sustainability. By obtaining stakeholder backing, the intervention aims to establish an environment in which WID can access necessary health services without experiencing stigma or fear.

##### Vision and mission

The vision of the Strategic Advocacy Activity is to achieve equitable access to health and harm reduction services for WID through consistent institutional and policy support. Its mission is to secure stakeholder commitment to protect the rights of WID, reduce stigma, and reinforce the policy and implementation landscape for effective harm reduction programming.

##### Program objectives

The program has four core objectives:

1. To secure stakeholder buy-in and support for harm reduction programs to ensure their sustainability and effectiveness.
2. To ensure protection of the rights of WID during service delivery.
3. To influence policies that facilitate service access for WID and support their socioeconomic well-being.
4. To align all harm reduction interventions with national guidelines, ensuring consistency and standardization.

##### Stakeholders analysis

The program requires the active involvement of multiple stakeholders. WID are central as primary beneficiaries. Religious and traditional leaders influence societal perceptions and are instrumental in shaping community support. Families of WID serve as critical support networks. Government institutions including the FMOH, SMOH, and law enforcement agencies are essential for both health service provision and legal protection. NASCP and SASCP ensure that the intervention aligns with national HIV/AIDS policy frameworks. NACA and SACA provide strategic oversight, while the National Technical Working Group (NTWG) offers technical direction. Nigeria Network of People Who Use Drugs (NNPUD) advocates for the interests of WID, and implementing partners and CBOs deliver operational support and local implementation.

##### Program steps

The program begins with the identification of advocacy issues and mapping of target audiences. This step will span two weeks and will culminate in a detailed problem analysis report. Subsequently, a one-month period is allocated for the development of an advocacy brief, including defined strategies and messaging frameworks. The finalized version of the advocacy brief, incorporating documented success stories, will be completed within an additional three weeks. The final stage involves scheduling quarterly advocacy visits by issuing stakeholder notifications and confirming meeting dates.

##### Monitoring and evaluation

Monitoring will track both implementation and influence. Key outputs include the number of advocacy visits, briefs developed, and stakeholder meetings. Outcomes will focus on stakeholder commitments, policy changes influenced, and the integration of harm reduction into institutional plans. Data will be gathered through event records, stakeholder feedback, and policy reviews.

##### Program funding sources

Initial and continued funding will be sourced from donors and government institutions to support program implementation and operational needs.

##### Financial sustainability plan

Advocacy efforts will be integrated into community-level initiatives. State and national TWGs will institutionalize advocacy activities to maintain harm reduction as a policy and program priority, independent of short-term funding cycles.

##### Program risks

Identified risks include stakeholder disengagement, restricted access to key decision-makers, and disruptions due to changes in government personnel.

##### Sustainability

Sustainability will be addressed through long-term engagement and capacity building of stakeholders to maintain consistent support for harm reduction. Embedding advocacy functions within state and national TWGs will provide institutional continuity and operational stability, even amid administrative or funding changes.

#### Human rights social campaigns intervention

##### Background

The Human Rights Social Campaigns Intervention addresses systemic human rights violations and gender-based violence experienced by WID. These violations, perpetrated by both law enforcement agencies (LEAs) and community members, contribute to an environment of fear and exclusion that hinders access to essential health services. The intervention aims to educate LEAs and the general public on the rights of WID and promote harm reduction principles. Campaign efforts will be anchored to global commemoration days to raise awareness and advocate for the rights and dignity of WID.

##### Program justification

Raising awareness among WID, LEAs, and the general public is necessary for improving the legal and social environment in which WID live. Education efforts will inform WID about their legal rights and available mechanisms for redress. Sensitizing LEAs is expected to shift institutional practices toward recognizing WID as rights-holders and populations at greater risk of harm rather than criminal offenders. Public education activities will address stigma at the community level and support increased access to harm reduction services.

##### Vision and mission

The vision of the Human Rights Social Campaigns Intervention is to establish a society where WID are aware of their rights and protected from abuse by law enforcement and the public. Its mission is to foster a legal and social framework that upholds the rights of WID, mitigates stigma, and facilitates access to harm reduction services through consistent public education and advocacy.

##### Program objectives

The intervention aims to:

1. Empower WID with knowledge of their legal rights and available protections.
2. Educate LEAs on harm reduction principles and the human rights context of WID.
3. Increase public awareness of the structural and social challenges faced by WID to foster broader community support for harm reduction.

##### Stakeholder analysis

The primary stakeholders include WID, who are the core beneficiaries of the intervention. LEAs are key actors due to their direct role in enforcement practices that affect WID. Community leaders—including religious figures and bunk owners^1^—exert significant influence over public attitudes and are critical for shaping a less stigmatizing and more supportive environment. Their inclusion is essential for advancing collaboration and ensuring effective implementation.

##### Program implementation

The campaign will produce tailored information, education, and communication (IEC) materials targeting both LEAs and WID. These will include posters, jingles, and electronic content to ensure accessibility and wide dissemination. Awareness activities will be conducted in alignment with major global commemoration days such as World Drug Day, World AIDS Day, and the 16 Days of Activism Against GBV. Public rallies, training workshops, and stakeholder engagement meetings will reinforce the campaign’s educational and advocacy objectives.

##### Monitoring and evaluation

Evaluation will cover campaign reach and impact. Outputs include the number of IEC materials produced, awareness activities conducted, and stakeholder participation. Outcomes will assess changes in LEA and community attitudes, reductions in reported stigma or abuse, and shifts in enforcement practices. Data will be collected through surveys, event records, and stakeholder feedback.

##### Program funding and sustainability

Program funding will be obtained from implementing partners, donor agencies, and government institutions. Sustainability will be supported through integration into government-led initiatives, inclusion in budget allocations for harm reduction awareness, and formalized partnerships with state-owned media for continued dissemination of campaign messages.

##### Program risks and mitigation strategies

Identified risks include limited funding and resistance from LEAs due to entrenched drug criminalization policies. Mitigation strategies involve sustained high-level advocacy to advance supportive policy reforms and secure governmental commitment to ongoing LEA sensitization and capacity-building efforts.

#### Training and Supportive Supervision (SSV) at the community level

##### Background

This intervention targets critical gaps in healthcare service delivery for WID, particularly in the areas of wound management and naloxone administration. The rise in untreated injection-related wounds and resulting amputations is compounded by a shortage of trained outreach workers (ORWs). Many existing ORWs lack the technical skills to deliver appropriate care, limiting the accessibility and quality of services available to WID. This intervention aims to equip ORWs with the required competencies to deliver essential healthcare services at the community level, in accordance with national guidelines, to reduce morbidity and mortality.

##### Program justification

Training ORWs in wound care and naloxone use will directly improve health outcomes for WID. The intervention supports national harm reduction and healthcare priorities by expanding access to competent community-level care. Strengthening ORW capacity through structured training and supervision will reduce preventable complications and fatalities associated with injecting drug use.

##### Vision and mission

The vision of the program is a health system in which all WID have access to quality, need-responsive healthcare services at the community level. Its mission is to build the capacity of ORWs to deliver essential clinical interventions, with ongoing support and oversight to maintain standards and improve health outcomes.

##### Program objectives

The objectives of the intervention are:

1. To reduce morbidity and mortality rates among WID by improving access to timely and competent healthcare.
2. To train ORWs in wound care and naloxone administration based on national guidelines.
3. To institutionalize supportive supervision mechanisms that ensure quality assurance and continuous performance monitoring at the community level.

##### Stakeholder analysis

Primary stakeholders include WID, as the main beneficiaries, and ORWs who serve as the frontline providers of care. Community leaders influence acceptance and implementation at the local level. Government agencies such as the FMOH, SMOH, and NACA will provide policy guidance and regulatory oversight. Implementing partners will support training and operational activities, while law enforcement agencies will contribute to maintaining a secure and enabling environment for service delivery.

##### Program implementation

Initial implementation activities will include the mapping of CBOs and local service providers to identify partners for training and deployment. Qualified trainers will be selected and prepared to deliver competency-based sessions to ORWs. A standardized curriculum will be developed and used in both initial and cascade (step-down) training sessions to ensure consistency and scale. ORWs will be subject to regular supportive supervision and mentorship to reinforce skills, correct deficiencies, and sustain quality.

##### Monitoring and evaluation

Monitoring will assess training delivery and service outcomes. Outputs include the number of ORWs trained, supervision visits conducted, and training sessions held. Outcomes will track the number of WID receiving wound care, naloxone use by ORWs, and adherence to clinical standards. Data sources include training logs, supervision reports, and service records.

##### Program funding and sustainability

Financial support will be secured from donor organizations and government agencies. To ensure long-term sustainability, the intervention will be embedded within existing state-level healthcare monitoring structures. Ongoing ORW training and supervision will be supported through formal government engagement and resource allocation.

##### Program risks and mitigation strategies

Identified risks include naloxone supply shortages, lack of stakeholder engagement, and inconsistent training delivery. Risk mitigation strategies include policy advocacy for reliable naloxone access, early engagement of key stakeholders to build commitment, and implementation of a structured training and supervision framework to standardize quality across all implementing entities.

### Empowerment and harm reduction interventions

#### HELP CARD Intervention

##### Background

The HELP CARD intervention addresses gender-based violence (GBV) and human rights violations experienced by WID. These violations occur within the people who inject drugs (PWID) community and are also perpetrated by law enforcement personnel, creating an environment of fear and discouraging WID from accessing essential health and social services. The HELP CARD is a physical resource that contains a directory of local service providers and outreach workers, including their contact information and availability. The lack of effective reporting mechanisms and the absence of trust in law enforcement are major barriers to service uptake. This intervention is designed to bridge this gap by enabling safe and direct contact between WID and service providers, ensuring timely response to reports of GBV and human rights abuses.

##### Program justification

Current systems for addressing GBV among WID are fragmented and inaccessible. The HELP CARD provides a structured, low-barrier mechanism to facilitate access to legal, medical, and psychosocial support services. By offering a vetted directory of outreach workers and legal aid providers, the intervention aims to improve response efficiency and reporting accuracy. It supports national harm reduction objectives by increasing service visibility and informing WID of their rights and support options.

##### Vision and mission

The vision is to establish a community where WID live free from GBV and human rights violations. The mission is to provide accessible reporting and referral channels that allow WID to report violations without fear of retaliation or further harm.

##### Program objectives

1. To increase the reporting and referral of GBV and human rights violations among WID.
2. To reduce the overall incidence of violence against WID.
3. To improve access to legal, health, and psychosocial services.
4. To enhance coordination between service providers and the WID community.

##### Stakeholder analysis

WID are the primary users of the HELP CARD. Outreach workers and service providers listed on the card serve as frontline responders. Implementing partners (IPs) facilitate coordination, ensure service availability, and manage the HELP CARD database. Law enforcement agencies are responsible for ensuring the safety of WID and responding to reported cases. The National Human Rights Commission (NHRC) oversees legal compliance. Legislative bodies provide policy support and help institutionalize the intervention for long-term impact.

##### Program implementation

Implementation will begin with a baseline assessment to identify and validate outreach workers and service providers. Funding for card production and distribution will be secured from implementing partners and donors. Stakeholder meetings will be conducted with WID, NHRC, NACA, IPs, law enforcement, and other relevant actors to define implementation roles. Contact information for service providers will be compiled by local government area, and the HELP CARD will be printed and distributed among WID. The use of the cards for referrals and reports will be monitored continuously to assess effectiveness.

##### Monitoring and evaluation

The program will be monitored using the following indicators: number of stakeholder engagement meetings conducted; number of HELP CARDs printed, distributed, and received by WID; and number of GBV and human rights cases reported and referred using the HELP CARD. Monitoring data will be used to evaluate the intervention’s effectiveness in increasing access to services and improving the reporting process.

##### Program funding and sustainability

Funding will be sourced from implementing partners and relevant government agencies. Long-term sustainability will be supported by bi-annual budget allocations from the Federal Ministry of Health (FMOH), NACA, and other stakeholders for card updates and reprinting. Integration into existing harm reduction programs will ensure ongoing relevance and reach.

##### Program risks and sustainability

The primary risk is the potential for retaliation against WID who report GBV, either from within the PWID community or from law enforcement actors. To mitigate this, confidentiality will be prioritized, and secure reporting protocols will be established. Sustainability will be achieved through integration into the Drug Demand and Harm Reduction Unit of the FMOH, with institutional support from NASCP, NHRC, and law enforcement agencies. This approach ensures continuity regardless of changes in funding or implementing partners.

#### SPEAK OUT! Intervention

##### Background

The SPEAK OUT! intervention addresses GBV and human rights abuses encountered by WID, with risks stemming from both the PWID community and law enforcement agencies. The intervention is designed to provide a cost-effective and accessible mechanism for WID to report abuses and access redress.

##### Program justification

An accessible and efficient system for reporting and seeking redress is critical for addressing GBV and human rights abuses among WID. The SPEAK OUT! initiative responds to this need by offering a streamlined platform through which WID can report incidents and access support in a timely and cost-effective manner.

##### Vision and mission

The vision is to build an empowered WID community equipped with reliable communication channels for reporting GBV and human rights violations. The mission is to utilize effective communication strategies to reduce WID’s vulnerability to such abuses and to improve their access to redress mechanisms and legal protections.

##### Program objectives

1. To protect WID from GBV and human rights abuses through improved access to reporting and redress mechanisms.
2. To establish and maintain accessible, user-friendly communication channels for abuse reporting.
3. To ensure timely and appropriate responses to reported cases of abuse.
4. To strengthen institutional capacity for handling reports and providing support to WID.

##### Stakeholders’ analysis

Primary users of the system are WID. Government agencies including NHRC, NACA, and FMOH are responsible for owning and managing the reporting and support platforms. Implementing partners connect these agencies with WID and support coordination with donors. Media outlets (TV, radio, print) are involved in awareness campaigns. Technical implementation depends on IT and telecommunications service providers such as MTN and Airtel, which support the digital infrastructure for the intervention.

##### Program steps

The program will begin with high-level advocacy visits to key stakeholders to secure institutional support over a period of 2 to 4 weeks. Stakeholder mandate meetings will follow over 4 to 6 weeks to align contributions. The identification and collation of implementing partners and service providers responsible for responding to GBV and rights violations will occur over an initial 4-week period and continue as needed. These service providers will be onboarded into the database for the mobile application, call centers, and SMS responders during a 2-week phase. Recruitment and engagement of focal persons to serve as first-line responders for calls and messages will take 4 to 8 weeks. Awareness and sensitization activities will be carried out over 4 to 6 weeks using media and town hall meetings and will continue as needed. Training for platform operators on technical operations and ethical conduct will be conducted over 2 to 4 weeks, while end-user training and testing will follow over 4 to 6 weeks.

##### Key milestones and deliverables

Milestones include the completion of advocacy visits and stakeholder meetings, compilation of a service provider list disaggregated by LGA, and onboarding of operators and end-users. Deliverables include the number of service providers engaged, quantity and reach of media promotions, the number of support group meetings where SPEAK OUT is introduced, and usage data from WID. The intervention will also track the number of successful referrals and documented cases of GBV and rights violations resolved through the system.

##### Monitoring and evaluation

Monitoring will be based on documented stakeholder engagement activities, the number of service providers included in the system, and the volume and reach of promotional campaigns. Additional metrics include support group engagement, number of operator and end-user training sessions, and platform usage statistics. Evaluation will also measure referral completion rates and the volume of cases addressed via the SPEAK OUT platform.

##### Program funding

Financial support for the initiative will come from NHRC budgetary allocations and partnership agreements between NACA and telecommunications firms. Long-term funding stability will rely on continued contributions from both government bodies and private-sector telecom providers.

##### Program risks and sustainability

Risks include potential breaches of confidentiality and retaliation against WID reporting abuse. Sustainability will be supported through government ownership and endorsement by security agencies. Institutional support will be critical to maintaining the intervention in the face of operational or funding disruptions.

#### Harm reduction education for WID

##### Background

The harm reduction education for WID intervention addresses the specific vulnerabilities of WID and their children by delivering targeted education and support. General harm reduction programs often neglect the gender-specific needs of WID, requiring a tailored approach. This intervention is designed to equip WID with the knowledge and tools to improve their safety and social integration. It aligns with global strategies that emphasize the inclusion of marginalized and high-risk populations.

##### Program justification

Existing drug use interventions do not account for gender-specific needs, thereby excluding WID from essential services. Biologically, women are more susceptible to infections such as HIV and Hepatitis. Nigeria, as a signatory to the global AIDS strategy, is committed to addressing the needs of all priority populations. Meeting the specific needs of WID is essential for achieving equitable access to care and for fulfilling international and national health commitments.

##### Vision and mission

The vision is to establish a society where WID have the knowledge and support to engage in safer drug use and access health and social services without stigma. The mission is to provide gender-sensitive education that promotes safer practices, skill development, and increased utilization of harm reduction services among WID.

##### Program objectives

1. To equip WID with the knowledge and skills necessary for improved social functioning
2. To reduce reported child neglect cases among WID households
3. To ensure WID have consistent access to harm reduction and hygiene products.

##### Stakeholders analysis

Key stakeholders include WID, their partners and children, relevant MDAs including law enforcement bodies, UN agencies, development partners, CBOs, and community and religious leaders. Each stakeholder group plays a role in service delivery, advocacy, and community engagement.

##### Program steps

The program will commence with proposal drafting note over a 2-week period, detailing the intervention’s objectives, methodology, and outcomes. Resource mobilization will follow over 6 months, including the procurement of financial, human, and material resources. Training materials tailored to the needs of WID will be developed in a 3-week phase. A one-week training session will be conducted to build the interpersonal communication (IPC) capacity of WID. IPC implementation will proceed using peer-to-peer sessions, focus group discussions, and support groups over a one-year period. Concurrent monitoring activities will assess IPC delivery and effectiveness using structured tools. At the end of the first year, a comprehensive evaluation will be conducted to assess the program’s impact, effectiveness, and areas requiring improvement. The resulting evaluation report will inform program adjustments and provide guidance for long-term sustainability.

##### Monitoring and evaluation

Monitoring will involve tracking the number of WID reached with IEC materials and the number participating in at least one IPC session. Additional indicators include session quality, level of support group engagement, and changes in safe drug use practices. Evaluation will focus on outcomes related to behavior change, knowledge retention, and access to harm reduction services.

##### Program funding

Funding can be sought from UN-Women, UNICEF, PEPFAR, UNDP, GF, WHO, UNODC, the Government of Nigeria, and domestic sources such as religious philanthropists and community organizations. These resources will support all phases of service delivery, training, and material provision. To ensure long-term sustainability, the program will be integrated into federal and state government frameworks with dedicated budget lines and policy support, reducing dependence on external donors.

##### Program risks and sustainability

Identified risks include potential funding gaps and concerns about confidentiality or reprisals from law enforcement or PWID community members, which may deter participation. Mitigation strategies include strong advocacy for government ownership and program institutionalization. Collaboration with law enforcement agencies will promote confidentiality and trust, while legal safeguards and continuous advocacy will support long-term sustainability.

### Integrated support and health interventions

#### Multi-pronged approach in empowering WID intervention

##### Background

This intervention is designed to address the compounded vulnerabilities faced by WID and their children through coordinated harm reduction, vocational training, psychosocial support, and child protection strategies. Existing gender-neutral interventions for people who use drugs often overlook the specific needs of WID, contributing to their increased exposure to HIV, Hepatitis, and associated social risks. This initiative aims to fill these service and structural gaps by delivering tailored support and engaging both governmental and community systems to ensure sustained access to comprehensive services.

##### Justification

Addressing the specific vulnerabilities of WID is essential to fulfilling Nigeria’s commitment under the global AIDS strategy. Failure to incorporate gender-responsive interventions undermines public health goals and perpetuates inequities in service delivery. Comprehensive and targeted support for WID is necessary to reduce these disparities and achieve effective inclusion.

##### Vision and mission

The intervention envisions an integrated system where WID benefit from structured harm reduction and empowerment services that improve their health and social outcomes. Its mission is to deliver targeted, evidence-informed programs that address the intersecting challenges faced by WID, through coordinated engagement with government bodies, community actors, and implementing partners.

##### Program objectives

1. To improve the socio-economic functionality of WID through sustained vocational and psychosocial interventions.
2. To reduce vulnerability of children of WID to exploitation and neglect.
3. To ensure uninterrupted access to harm reduction commodities and hygiene supplies for WID.

##### Stakeholders

Key stakeholders in this intervention include WID, their partners and children, relevant government ministries, departments and agencies, law enforcement institutions, development partners, community-based organizations, and traditional and religious leaders. These actors are expected to contribute to the planning, implementation, and monitoring of activities to ensure relevance, coordination, and accountability.

##### Program components

The program will begin with the development of an advocacy brief detailing the rationale for skills training and equipment support for WID. This document will be presented to selected government agencies, followed by structured advocacy visits and a defined period of engagement to track commitments and follow-through. Training activities will involve the creation of training materials, selection and preparation of master trainers, and the implementation of a six-month structured skills acquisition program for WID, who will also be trained to serve as peer mentors. The establishment of safe spaces and vocational centers will proceed in phases, beginning with resource mobilization, site identification, and procurement, followed by operationalization of training hubs. Personal hygiene interventions will include the development of sensitization materials, procurement of hygiene products, and a structured distribution system supported by routine educational sessions. All program components will be subjected to ongoing internal monitoring to assess implementation fidelity, service uptake, and short-term outcomes.

##### Monitoring and evaluation

The program will track quantitative and qualitative indicators to measure both output and outcome-level achievements. This includes the number of WID trained and retained as mentors, the number of active skills centers and safe spaces, and the quantity and frequency of hygiene product distribution. Additional monitoring efforts will assess beneficiary satisfaction, the perceived impact of services on daily functioning, and the extent to which children of WID are protected through related program linkages. These indicators will inform real-time adjustments and support evidence-based decision-making throughout the life of the intervention.

##### Funding strategy

Funding will be sourced from multilateral organizations including UN-Women, UNDP, GF, WHO, and UNODC, alongside national sources and community-level actors such as religious philanthropists and local organizations. To ensure sustainability, the program will pursue formal integration into existing national and subnational government programs. A phased transition plan will be developed, outlining the timeline and conditions for full government ownership of all program components.

##### Risks and sustainability

The primary risks to implementation include the potential discontinuation of donor funding and the diversion of distributed supplies such as hygiene products. To address these risks, the program will engage government institutions early to secure long-term budgetary allocations. Distribution systems will be designed with traceability mechanisms to prevent diversion, and security agencies will be involved in monitoring where appropriate. These measures will support the operational integrity and continuity of the intervention, even in the absence of external funding.

#### Social protection for WID and their children intervention

##### Background

This intervention aims to reduce the social and health-related vulnerabilities of WID and their children by strengthening access to social protection services. Existing programs targeting PWUDs are generally not gender-responsive, resulting in the systemic exclusion of WID from essential services. Their children, due to their caregivers' precarious socio-economic and health conditions, also face significant risks. The intervention proposes the integration of WID into health insurance schemes, improved access to child protection services, and structured nutritional support, aligned with national commitments under the global AIDS strategy to provide inclusive and equitable service coverage.

##### Justification

WID constitute a distinct subpopulation with specific vulnerabilities that are not addressed by general PWUD-focused interventions. Inclusion in social protection systems, especially in the areas of health coverage, legal identity, and basic nutrition, is essential for reducing structural disadvantages and improving health and social outcomes for WID and their children.

##### Vision and mission

The vision of this intervention is a social system where WID and their children are adequately protected through access to structured, equitable, and responsive social support services. Its mission is to implement mechanisms that provide WID with health insurance, protect the rights and well-being of their children, and ensure consistent access to basic nutrition and legal documentation.

##### Program objectives

1. To improve the capacity of WID to participate more effectively in society.
2. To ensure that children of WID are protected from exploitation and social neglect.
3. To guarantee access for WID to essential health and social protection services.

##### Stakeholders

Primary stakeholders include WID and their families, relevant government MDAs, law enforcement agencies, development partners, CBOs, and religious and community leaders. These actors are expected to support planning, advocacy, implementation, and monitoring efforts to ensure contextual appropriateness and effectiveness.

##### Program components

The intervention will begin by facilitating the inclusion of WID in national and state health insurance schemes. This will be operationalized through the development of a concept note, stakeholder engagement for resource mobilization, and enrolment support. Enrolled individuals will be monitored for one year to evaluate uptake and continuity, while parallel advocacy efforts will target the inclusion of WID in government insurance planning frameworks to ensure long-term sustainability.

To address health-related queries and access barriers, the program will leverage the existing 6222 helpline. Advocacy visits to NACA will be conducted to incorporate WID-specific response protocols into the system. Standard operating procedures will be developed for helpline staff, and the responsiveness to WID will be monitored over a 12-month period to identify gaps and improve service quality.

Legal protection of children will be supported through advocacy for the domestication and enforcement of the Child Rights Act in applicable states. This will involve the preparation of advocacy materials, stakeholder sensitization, and a 24-month engagement period to track legislative and policy progress. For improved civil registration, a WID-specific child database will be developed and supported by awareness campaigns and structured registration drives. Monitoring will assess the number of birth and death registrations completed and integrated into national databases.

Nutritional support will be delivered through a structured food voucher system. Program activities will include concept note development, resource mobilization, and the integration of voucher distribution into existing nutritional programs. Implementation will be monitored for one year to assess impact and refine delivery mechanisms.

##### Monitoring and evaluation

Monitoring will focus on both service coverage and outcome-level indicators. These will include the number of WID enrolled in health insurance schemes, the volume and quality of responses to WID through the helpline, the number of advocacy engagements related to child protection legislation, and the number of birth certificates issued to children of WID. The number of WID accessing nutritional support will also be tracked. Data will be analyzed to inform adaptive planning and ensure that intervention targets are met efficiently.

##### Funding strategy

Funding will be pursued through partnerships with UNICEF, UN-Women, UNDP, and GF, as well as through national allocations and local contributions. The intervention will be designed for integration into existing state-level social protection programs to ensure sustainability. Formal transition planning will be initiated early to secure long-term financial and institutional commitment by relevant government entities.

##### Risks and sustainability

Program risks include limited fiscal space at the government level, inconsistent donor support, low political will to prioritize WID, and limited engagement from WID themselves due to stigma or legal fears. These risks will be addressed through early institutional engagement, stakeholder mapping, and community mobilization strategies tailored to the needs and concerns of WID. Long-term sustainability will be pursued by embedding all activities within existing government systems, ensuring technical and budgetary continuity even in the absence of external funding.

#### Subsidized cost of services

##### Background

This intervention addresses the financial barriers that limit WID’s access to essential health services. High out-of-pocket costs prevent many WID from obtaining Hepatitis B virus (HBV) vaccination, Hepatitis C virus (HCV) treatment, cervical cancer screening, and wound care services. These barriers contribute to poor health outcomes and elevate the risk of preventable disease transmission within and beyond this population. Subsidizing the cost of these critical services will increase service uptake and improve overall health indicators among WID.

##### Justification

Subsidizing the cost of essential services is a necessary step toward achieving equitable access for WID. This population is not only medically vulnerable but also economically disadvantaged, which makes them disproportionately affected by high service costs. Reducing or removing this financial barrier aligns with national and global strategies to ensure that no population at greater risk of harm is excluded from lifesaving health services.

##### Vision and mission

The vision of the intervention is a health system where WID can access essential preventive and treatment services without financial hardship. The mission is to operationalize a cost-subsidization mechanism for key health interventions, ensuring affordability, increasing service utilization, and improving health outcomes among WID.

##### Program objectives (by 2030)

1. To increase the number of WID accessing HBV vaccination and HCV treatment.
2. To reduce the incidence and progression of HBV and HCV among WID.
3. To improve access to cervical cancer screening and wound care services for WID.

##### Stakeholders

Stakeholders include WID and their families, NASCP, SASCP, NACA, SACA, NTWG, NNPUD, implementing partners, donors, and CBOs. These actors are responsible for financing, service delivery, monitoring, and policy alignment to ensure effective and efficient implementation.

##### Program components

The program will begin with the identification of high-priority health services required by WID, followed by an assessment of associated costs across different geographical and service delivery contexts. This activity will be completed within one month and will produce a detailed report outlining service gaps and cost estimates. Implementing partners will then be identified for each service area, followed by a facility mapping exercise to ensure that services are available and geographically accessible to WID. A cost analysis will be conducted over two weeks to determine appropriate subsidy levels, resulting in a pricing framework for subsidized services. Funding sources will be identified and documented in a funding source database updated quarterly. This will guide proposal development and funding applications, which will be submitted on a rolling basis to maintain financial inflows. Once funding is secured, the subsidy program will be launched with routine monitoring of service delivery and client uptake.

##### Monitoring and evaluation

The program will track service utilization metrics, including the number of WID receiving HBV vaccinations, accessing HCV treatment, undergoing cervical cancer screening, and receiving wound care. Affordability will be assessed through beneficiary feedback and payment tracking. Additional indicators will include geographic coverage of participating facilities and timeliness of service delivery. Collected data will inform mid-course corrections and final program evaluation.

##### Funding strategy

Funding will be pursued from multilateral donors, the Government of Nigeria, and private foundations. Grant applications will be aligned with disease-specific funding mechanisms, such as those for HIV, viral hepatitis, or cancer prevention. A financial sustainability plan will be developed concurrently to ensure transition into domestic funding streams. Government ownership will be prioritized through phased integration of subsidized services into the national and state healthcare budgets.

##### Risks and sustainability

Key risks include inadequate stakeholder engagement, insufficient or short-term funding, and premature withdrawal of donor support. These will be addressed through early engagement with implementing stakeholders, diversification of funding sources, and the development of contingency financing plans. Sustainability will be pursued through formal adoption of subsidized service components into government health financing structures, ensuring continuity even in the absence of external support.

## Discussions

In this discussion, we highlight a few points that program implementers in WID programs need to be conversant for successful programming. Firstly, an integrated approach and multi-pronged strategy are important for addressing the complex vulnerabilities faced by WID. This approach, as demonstrated by the proposed interventions, combines advocacy, skills training, safe spaces, health insurance, and nutritional support to create a holistic framework. Such comprehensive strategies are necessary given the multifaceted challenges that WID encounter, including stigma, health risks, and social marginalization. Literature supports this integrated model since single-intervention approaches often fail to address the interconnected issues that exacerbate the vulnerability of WID [23]. Government and community engagement are also crucial components, as securing commitment and resources from these stakeholders ensures the sustainability and effectiveness of the interventions. Previous studies have also shown that programs that actively involve local communities and government agencies are more likely to succeed, as they are better aligned with local needs and have greater access to resources [24, 25]. Thus, the multi-pronged strategy not only addresses the immediate needs of WID but also builds a supportive environment for long-term change.

The specific interventions developed, such as empowerment through skills acquisition, social protection initiatives, and subsidized healthcare, are critical for addressing the unique needs of WID. Empowerment and skills training are vital for improving the societal integration of WID, as they equip individuals with the tools necessary to gain financial independence and reduce reliance on high-risk behaviors associated with drug use. This aligns with existing literature, which underscores the effectiveness of skills training programs in reducing vulnerability among marginalized populations by enhancing their economic stability and self-efficacy [26]. Social protection measures, including health insurance and nutritional support, play a significant role in safeguarding the well-being of WID and their children, who are often neglected in traditional harm reduction programs. These interventions are crucial for reducing health disparities as they ensure that WID have access to essential services that address both their physical and social needs [27]. Finally, subsidized healthcare services directly tackle the financial barriers that prevent WID from accessing necessary medical care, which is a well-documented obstacle in healthcare utilization among priority populations [28]. Addressing these specific areas not only improve the immediate health outcomes of WID but also contribute to long-term societal benefits by reducing the burden on public health systems.

Appropriate program objectives and metrics are foundational to the success of interventions targeting WID. This is because they provide clear goals and measurable indicators to assess progress. The programs' objectives are aligned with global health strategies, which are aimed at reducing vulnerabilities among marginalized populations[29]. These objectives include improving societal integration, protecting children from exploitation, and ensuring access to necessary health services. By setting clear targets, the programs described in this study establishes a long-term vision that is crucial for sustained impact. The monitoring and evaluation components of the programs allow program implementers to track key metrics. Such metrics include the number of WID trained, enrolled in health insurance, and reached with sensitization materials. These metrics are not only indicators of the program's reach but also its effectiveness in achieving desired outcomes [30]. The emphasis on continuous evaluation ensures that the program remains responsive to the needs of WID, allowing for data-driven adjustments and improvements over time. It has been established in literature that programs with robust monitoring and evaluation frameworks are more likely to succeed because they provide ongoing insights that can inform strategic decisions and resource allocation [31].

Funding and sustainability of interventions for WID are vital to the life span, viability, and success of these programs. Securing diverse funding sources, including international organizations, government agencies, and local donors, is essential for establishing a stable financial base that can support the wide range of services required by WID [32]. This approach not only alleviates the risk of reliance on a single funding stream but also aligns with best practices in program sustainability. Program sustainability advocates for a multi-source funding model to ensure resilience against financial disruptions [33]. Current funding prospects in Nigeria include (i) multilateral HIV streams (e.g., Global Fund HIV components, Unitaid) accessed through national grant consortia and principal recipients,(ii) bilateral HIV programmes (e.g., PEPFAR) channelled via implementing partners; and (iii) targeted philanthropy (e.g., ViiV Healthcare Positive Action) for focused packages such as gender-responsive harm reduction or community engagement. National implementing partners (e.g., the Center for Integrated Health Programs (CIHP) and Society for Family Health (SFH), among others) are plausible conduits for sub-awards and technical support.

Financial sustainability will be further strengthened by integrating the program into existing government frameworks. This strategy can help institutionalize the interventions and make them part of standard public health services. Piot and Quinn [34] emphasized the importance of government ownership in sustaining health interventions, particularly in low-resource settings, where external funding may fluctuate. Securing long-term commitment from stakeholders, will help programs maintain their operations beyond the initial funding period, ensuring continued support for WID. Relevant government ministries departments and agencies like NACA and NASCP continue to provide policy guidance, partner coordination, and integration into national and state plans, with the possibility of future budget lines if activities are codified in annual operational plans. Additionally, ongoing advocacy and stakeholder engagement are crucial for keeping the program's priorities aligned with national health agendas, thereby enhancing its sustainability.

Furthermore, risks and mitigation strategies are important considerations for the successful implementation and sustainability of programs targeting WID. One of the primary risks identified is the potential lack of sustained funding, which could jeopardize the continuity of services and interventions [32]. Donor fatigue and shifting priorities among funding agencies are also common challenges that can disrupt program financing. It is thus important to diversify funding sources and secure long-term commitments from multiple stakeholders, including government entities, international organizations, and local donors, so as to mitigate these risks. Another noteworthy risk is the potential for program integrity issues, such as product diversion or inadequate buy-in from key stakeholders. Rigorous monitoring and evaluation processes, coupled with regular audits, can help mitigate these risks by ensuring transparency and accountability in program operations [31]. Additionally, engaging security agencies and community leaders early in the programs can help build trust and support, reducing the likelihood of resistance or sabotage from within the community or law enforcement. Proactive risk management strategies, including stakeholder engagement and robust monitoring frameworks, are critical for the sustainability and effectiveness of health interventions [34].

Lastly, stakeholder involvement is a vital factor that determines the success of programs aimed at supporting WID. The involvement of a wide range of stakeholders—including government agencies, community leaders, development partners, and the WID themselves—ensures that the program is both comprehensive and responsive to the specific needs of the target population [24]. Engaging stakeholders from the outset fosters a sense of ownership and accountability, which is essential for the program's sustainability. Programs with active stakeholder participation are more likely to be effective as they benefit from local knowledge, resources, and networks that can enhance program implementation and outcomes [33]. For example, collaboration with law enforcement and community leaders can help mitigate stigma and reduce barriers to service access for WID, while partnerships with healthcare providers ensure the availability of essential health services [31]. Moreover, involving WID in the program design and evaluation processes empowers them and ensures that the interventions are culturally sensitive and aligned with their lived experiences. This participatory approach not only improves program relevance and effectiveness but also builds trust and engagement within the community, which are crucial for long-term impact.

This study has a few limitations. First, the WID recruited for FGDs and IDIs may not be fully representative of the broader WID population in Nigeria. Women with unstable housing, recent incarceration, or without CBO contact may have been under-represented. Geographic coverage was limited to three states, which may constrain transferability. Second, power dynamics in mixed-stakeholder activities could have shaped idea generation. To mitigate these, we (i) triangulated insights across states, and (ii) conducted state and national validation sessions. Finally, the intervention designs were developed through a co-creation process with multiple validation steps, but implementation has yet to begin. Our next step involves conducting a formal end-user usability test of the materials and workflows to inform pilot implementation. 

## Conclusion

Using HCD across qualitative assessment, co-creation, and validation, we defined an implementation-ready, multi-component package for WID that integrates strategic communication, empowerment/harm-reduction, and health/social protection services. The package aligns with existing systems and specifies monitoring and financing pathways, offering a practical template for large-scale harm-reduction implementation in under-resourced settings.

## Data Availability

Not applicable (this manuscript does not include analysis of any primary or secondary datasets).

## Abbreviation

CBO: Community-based organization
FGD: Focus group discussion
FMOH: Federal Ministry of Health
HBV: Hepatitis B virus
HCD: Human-centered design
HCV: Hepatitis C virus
IDI: In-depth interview
IEC: Information, education and communication
IP: Implementing partner
IPC: Interpersonal communication
LEA: Law enforcement agency
MDAs: Ministries, departments and agencies
NACA: National Agency for the Control of AIDS
NASCP: National AIDS and STIs Control Programme
NHRC: National Human Rights Commission
NNPUD: Network of Nigerian People Who Use Drugs
NSP: Needle and syringe programme
NTWG: National Technical Working Group
OAT: Opioid agonist treatment
ORW: Outreach worker
SACA: State Agency for the Control of AIDS
SASCP: State AIDS and STIs Control Programme
SFH: Society for Family Health
SMOH: State Ministry of Health
SMS: Short Message Service
WID: Women who inject drugs

## Footnotes

1 "Bunk" in the context of drug-using communities in Nigeria designates not only the geographical localization of living spaces predominantly occupied by individuals engaged in drug injection practices but also implies a structured hierarchy within these areas. This hierarchical structure manifests in the organization and dynamics of the community, influencing aspects such as control over drug supply channels, allocation of resources, and decision-making processes. Individuals or groups at higher levels of this hierarchy may exert influence or control over those at lower levels, affecting access to drugs, living conditions, and safety within the community. This hierarchical organization can further exacerbate vulnerabilities among community members, leading to disparities in health outcomes, security, and social support. The term "bunk" thus encompasses the complexities of spatial, social, and economic factors that characterize the living environments of drug-using populations in Nigeria, highlighting both the physical geography and the embedded social structures.
